# Relationship between maternal exposure to heavy metal titanium and offspring congenital heart defects in Lanzhou, China: A nested case-control study

**DOI:** 10.3389/fpubh.2022.946439

**Published:** 2022-08-03

**Authors:** Jianhao Sun, Baohong Mao, Zhenzhen Wu, Xinjuan Jiao, Yanxia Wang, Yongli Lu, Xuejing Ma, Xiaohui Liu, Xiaoying Xu, Hongmei Cui, Xiaojuan Lin, Bin Yi, Jie Qiu, Qing Liu

**Affiliations:** ^1^Gansu Provincial Maternity and Child-Care Hospital, Lanzhou, China; ^2^The First Clinical Medical College, Gansu University of Chinese Medicine, Lanzhou, China; ^3^School of Nursing, Gansu University of Chinese Medicine, Lanzhou, China

**Keywords:** congenital heart defects, metal exposure, titanium, pregnancy, maternal blood, umbilical cord blood

## Abstract

**Background:**

Previous studies have found that exposure to heavy metals increased the incidence of congenital heart defects (CHDs). However, there is a paucity of information about the connection between exposure to titanium and CHDs. This study sought to examine the relationship between prenatal titanium exposure and the risk of CHDs in offspring.

**Methods:**

We looked back on a birth cohort study that was carried out in our hospital between 2010 and 2012. The associations between titanium exposure and the risk of CHDs were analyzed by using logistic regression analysis to investigate titanium concentrations in maternal whole blood and fetal umbilical cord blood.

**Results:**

A total of 97 case groups and 194 control groups were included for a nested case-control study. The [P_50_ (P_25_, P_75_)] of titanium were 371.91 (188.85, 659.15) μg/L and 370.43 (264.86, 459.76) μg/L in serum titanium levels in pregnant women and in umbilical cord serum titanium content in the CHDs group, respectively. There was a moderate positive correlation between the concentration of titanium in pregnant women's blood and that in umbilical cord blood. A higher concentrations of maternal blood titanium level was associated with a greater risk of CHDs (OR 2.706, 95% CI 1.547–4.734), the multiple CHDs (OR 2.382, 95% CI 1.219–4.655), atrial septal defects (OR 2.367, 95% CI 1.215–4.609), and patent ductus arteriosus (OR 2.412, 95% CI 1.336–4.357). Dramatically higher concentrations of umbilical cord blood levels had an increased risk of CHDs and different heart defects.

**Conclusion:**

Titanium can cross the placental barrier and the occurrence of CHDs may be related to titanium exposure.

## Introduction

Congenital heart diseases (CHDs) refer to congenital heart structure and function defects during embryonic development. As one of the most common birth defects, the global prevalence rate is about 0.8–1.2% (1). The quality of life for patients who have CHDs is reduced by the disease's high mortality and morbidity rates, which may necessitate several operations to fix problems (2). CHDs is caused by genetic factors, environmental factors alone or both (3). Compared with genetic factors, environmental factors are more recognizable and controllable, so CHDs can be prevented by identifying and avoiding adverse environmental risk factors.

With the development of modern society and the progress of industry level, the living environment and industrial pollution are increasing day by day, which seriously endangers the life and health of pregnant women and fetuses. Among them, heavy metal pollution is one of the main environmental pollution at present. Current studies indicate that maternal exposure to heavy metal during pregnancy is a risk factor for CHDs, including cadmium, arsenic, copper, barium and nickel (4, 5). Titanium (Ti) is one of the most common heavy metal elements. Among its common form, titanium dioxide (TiO_2_) can be used as coatings, plastics, personal care products (cosmetics, sunscreen) and food in the common additives (6). The European Chemicals Agency (ECHA) classifies TiO_2_ as a Category 2 carcinogens: suspected human carcinogen inhaled (7).

Titanium can be absorbed by the human body through ingestion, inhalation, skin media and implant dissolution (8). Numerous *in vivo* and *in vitro* studies have shown that exposure to titanium induces cytotoxicity, oxidative stress, and inflammation and disrupts DNA and lipid metabolism (9–11). Animal experiments implied titanium and titanium compounds have been known to cause neurotoxicity, reproductive toxicity, skeletal malformations, and cardiopulmonary effects in offspring (12). It was found that prenatal exposure to TiO_2_ NPs inhibited the expression of Rac1 and Cdc42 proteins in the offspring's hippocampus and reduced the number of axon and dendrite branches, possibly inhibiting the offspring's brain development (13). TiO_2_ nanoparticles (20 nm; 5 mg/mL, 21 nm; 0.01, 10 and 1,000 g/mL, and 240–280 nm in water; 0.1 g/mL) caused circulatory system abnormalities, premature hatching, and reduced reproduction in zebrafish in prior research (14, 15). Additionally, unborn mice's skeletal development fell behind that of the control group when their mother was exposed to 100 mg/kg nano-TiO_2_, leading to loss or reduction of ossification and fetal dysplasia. The interference of calcium, zinc and other metabolic processes by nano-tio2 may be the cause of these side effects, either directly or indirectly (16). In addition, following exposure to nano-TiO_2_, mice's decreased fertility and ovarian damage may be linked to altered production of cytokines related to inflammation or follicular atresia (17).

*In vivo* tests show that TiO_2_ will accumulate in the lungs, digestive tract, liver, heart, spleen, kidney and myocardium after inhalation or oral exposure (18). A growing number of epidemiological studies suggested that titanium exposure may be associated with diabetes, colitis, and cardiovascular disease (11, 19, 20). In addition, exposure to titanium was found to be associated with adverse pregnancy outcomes, including fetal distress, preterm birth, low birth weight and neural tube defects (8, 21, 22).

However, few studies have independently assessed the correlation between titanium concentrations in maternal whole blood and umbilical cord blood and the risk of CHDs in offspring. In order to thoroughly investigate the relationship between maternal titanium exposure and the risk of CHDs developing in offspring, we carried out a prospective nested case-control research in Lanzhou, China.

## Methods

### Study population

**Figure 1:**
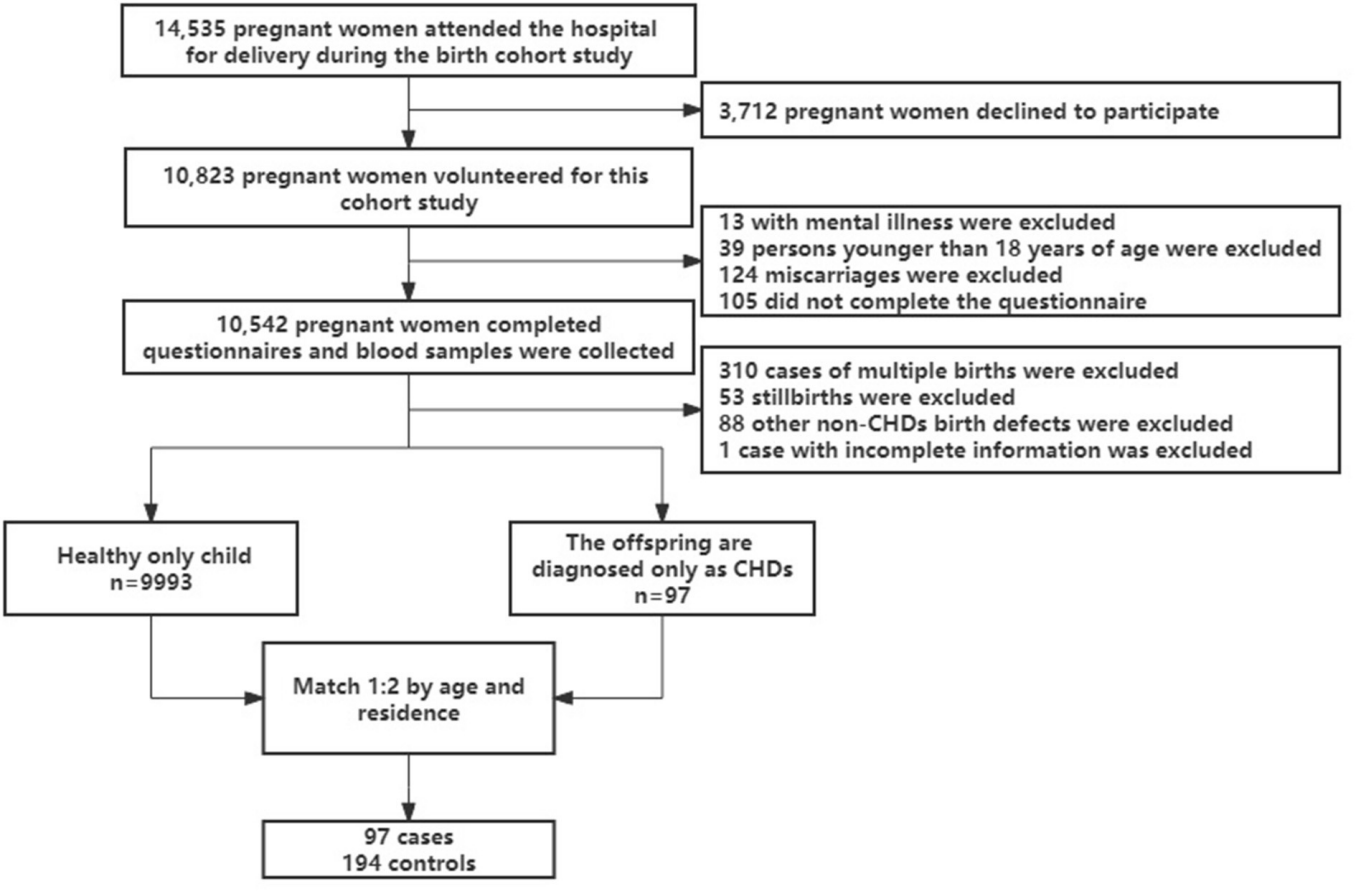
The flow chart of study population selection.

In Lanzhou, China, at the Gansu Provincial Maternity and Child Care Hospital, a birth cohort study was carried out between 2010 and 2012 (23). A total of 14,535 pregnant women delivered at the hospital during this time. Pregnant women who delivered at least 20 weeks pregnant, without mental illness, and aged at least 18 years were eligible, 176 of whom were not eligible for the study (13 with mental illness, 39 under 18 years of age, and 124 miscarried within 20 weeks). Fourteen thousand three hundred fifty-nine eligible women contacted, 3,712 declined to participate and 105 did not complete the questionnaire. Finally, a total of 10,542 people completed the baseline questionnaire and blood sample collection, with a response rate of 73.4%. Excluding multiple births, stillbirths and/or other birth defects (non-CHDs) and incomplete information at baseline, 97 of the only children born to the study subjects were diagnosed with CHDs. In this study, subjects who delivered a healthy only child at baseline were selected as controls, and subjects were randomly selected from the controls for 1:2 matching based on age (±2 years) and place of residence. A total of 291 subjects were included in the study (as showed in Figure 1). The research project was approved by the Medical Ethics Committee of the Gansu Provincial Maternity and Child Care Hospital and Yale University. When eligible women arrived at the hospital for the birth, they were briefed about the study's protocol. A systematic and structured questionnaire was used to conduct in-person interviews in the hospital following the receipt of written consent. The majority of women (84%) were questioned during the first 3 days following birth. Demographic data, reproductive and medical history, smoking habits, alcohol and tea use, employment and housing history, physical activity level, and food were all gathered *via* the questionnaire. The medical records were mined for data on pregnancy complications and birth outcomes.

### Classification of congenital heart defects

Depending on the degree of the problem, each congenital cardiac abnormality was classified as “Isolated,” “Multiple,” or “Syndrome.” Our previous study provided more detailed information on the classification of CHDs (24). All CHDs were divided into three categories based on the clinical phenotype and the ICD-10 codes (Q20–Q28) for the International Classification of Diseases: (1) Congenital abnormalities of the great arteries, such as patent ductus arteriosus (PDA), aortic coarctation, pulmonary artery stenosis, and other congenital malformations of the great arteries (ICD 10: Q25). (2) Congenital septal malformations, such as atrioventricular septal defects (AVSDs), ventricular septal defects (VSDs), atrial septal defects (ASDs), and other congenital cardiac septal malformations (ICD 10: Q21). (3) Other CHDs, such as congenital heart abnormalities, defects in the chambers and connections of the heart, etc.

### Blood collection, storage and analysis

The subjects blood samples were collected at the time of delivery in hospital. 3 ml of whole blood from the median elbow vein was collected on an empty stomach in the morning, and 7 ml of Ethylenediaminetetraacetic acid (EDTA) Vacutainer blood was collected 3 mL of cord blood was also taken before placental abruption (cesarean section or vaginal delivery) and collected with 7 mL special Vacutainer of EDTA for anticoagulation, and stored in a refrigerator AT-80 degrees Celsius. All sample information is incorporated into the electronic database of biological samples.

On an electric hot plate set at 175°C for 6 h, 0.2 mL of whole blood is combined with 1 mL HNO3 and 0.2 mL H2O2. Following digestion, deionized water is used to dilute the solution to a final volume of 10 mL. After that, inductively coupled plasma-tandem mass spectrometry is used to detect the amount of titanium in maternal and cord blood (ICP-MS, iCAP RQ; Thermo Fisher, USA). We employed the internal standard approach for quality control and chose the internal standards Sc45, Ge72, Rh103, and Re187. Measurements are made twice on each sample. We tested the standard reference materials during the experiment for every 20 samples, and we verified that the values of the measured reference materials were within the suggested range for each metal. Ti had a 0.22 g/L lower limit of detection (LOD). When the concentration of titanium was below the limit of detection, it was reported as zero.

### Statistical analysis

SPSS Statistics 25.0 was used to conduct all statistical analyses (IBM, Chicago, USA). Percentage was used to describe the classification variables of pregnant women, such as BMI, education level, family per capita monthly income, production time and smoking history. The case group and the control group's disparities in maternal traits and demographic data were compared using the chi-square test. Continuous variables, such as the level of titanium element in each biological sample, were not in accordance with normal distribution, so they were represented by median and quartile range. Wilcoxon-Mann-Whitney U test was employed to compare groups. In order to explore the correlation between titanium level and CHDs in offspring, Multivariate conditional Logistic regression was used to analyze the factors of CHDs caused by titanium exposure in pregnancy. *P* < 0.05 denoted a statistically significant difference and was used to include factors in a multivariate analysis that had *P* < 0.05 in the univariate analysis.

Since there is no normal reference range for maternal blood titanium level and umbilical cord blood titanium level, Receiver Operating Characteristic (ROC) curve was used to analyze the influence of maternal blood titanium level and umbilical cord blood titanium level on the occurrence of CHDs, with the occurrence of CHDs as the dependent variable, and the maternal blood titanium level and umbilical cord blood titanium level in the original data as the independent variable. We refer to previous research methods (25), the ROC curve was used to obtain cut-off values to classify maternal and umbilical cord blood titanium levels into high and low concentrations; Low concentrations in maternal and umbilical cord blood titanium levels were used as reference; Two-tailed *P* < 0.05 and 95% CIs excluding 1.00 were considered statistically significant.

## Results

### Characteristics of participants

One hundred ninety-four controls and 97 cases were recruited and all biological samples were obtained. The 97 cases of CHDs included The isolated CHDs (43 cases), The multiple CHDs (48 cases), The Syndrome CHDs (6 cases), PDA (70 cases), ASDs (48 cases), VSDs (7 cases) and AVSDs (4 cases). The maternal characteristics of the samples are shown in Table 1. Take vitamin or mineral supplements during the first 13 weeks of pregnancy and after 27 weeks of pregnancy were significantly different between the two groups (*P* < 0.01, In the control group, 52 women were deficient in vitamin use before 13 weeks of pregnancy), Other baseline data showed no significant difference between the two groups.

**Table 1:** Descriptive characteristics of the study sample.

**Variable**	**Cases** ***N* = 97 (%)**	**Control** ***N* = 194 (%)**	**Chi square**	***P*-value**
Maternal pre-pregnancy BMI (kg/m^2^)			3.191	0.354
BMI <18.5	16 (16.5)	37 (19.1)		
18.5–24	66 (68.0)	138 (71.1)		
BMI>24	15 (15.5)	19 (9.8)		
Maternal age (years, *n*)			3.034	0.082
*n* ≤ 35	80 (82.5)	174 (89.7)		
*n* > 35	17 (17.5)	20 (10.3)		
Chinese Han population			2.445	0.118
Yes	90 (92.8)	189 (97.4)		
No	7 (7.2)	5 (2.6)		
Educational level of pregnant women			0.838	0.658
Junior high or below	34 (35.1)	60 (30.9)		
Technical secondary school, high school	16 (16.5)	29 (14.9)		
Undergraduate college or above	47 (48.4)	105 (54.2)		
Monthly household income (CNY, *n*)			0.609	0.737
*n* <3000	57 (58.8)	107 (55.2)		
3,000–5,000	21 (21.6)	50 (25.8)		
*n* > 5000	19 (19.6)	37 (19.0)		
Work during pregnancy			0.062	0.901
Yes	47 (48.5)	91 (46.9)		
No	50 (51.5)	103 (53.1)		
Number of previous births (freq, *n*)			3.567	0.168
*n* = 0	68 (70.1)	129 (66.5)		
*n* = 1	21 (21.6)	57 (29.4)		
*n* ≥ 2	8 (8.3)	8 (4.1)		
Active/passive smoking during pregnancy			0.293	0.628
Yes	19 (19.6)	33 (17)		
No	78 (80.4)	161 (83)		
Alcohol consumption before pregnancy			0.129	0.719
Yes	4 (4.1)	5 (2.6)		
No	93 (95.9)	189 (97.4)		
Take vitamins or minerals before 13 weeks of pregnancy			41.959	<0.01
Yes	63 (64.9)	137 (96.5)		
No	34 (35.1)	5 (3.5)		
Take vitamins or minerals between 14 and 26 weeks of pregnancy			0.557	0.455
Yes	47 (48.5)	103 (53.1)		
No	50 (51.5)	91 (46.9)		
Take vitamins or minerals after 27 weeks of pregnancy			8.908	<0.01
Yes	36 (37.1)	108 (55.7)		
No	61 (62.9)	86 (44.3)		

### Concentration of titanium in blood samples from pregnant women

Compared with the control group, the level of titanium in the blood samples of pregnant women in the case group difference was statistically significant (*P* < 0.05), as showed in Table 2. The median titanium concentration in pregnant women was 304.65 μg/L, including the case group and the control group. We conducted a statistical analysis on the concentration of titanium in the blood of pregnant women with different CHDs subtypes. There were statistically significant differences between some subtypes in the case group and the control group, such as the isolated CHDs, The multiple CHDs and ASDs (*P* < 0.05).

**Table 2:** Comparison of serum titanium levels between pregnant women in case group and control group.

**Serum titanium levels in pregnant**						
**women [P**_**50**_ **(P**_**25**_**, P**_**75**_**)**, μ**g/L]**						
**Group**	**N**	**P_25_**	**P_50_**	**P_75_**	**Z**	***P*-value**
Control	194	217.34	302.41	389.85		
Case	97	188.85	371.91	659.15	−2.485	0.013
The isolated CHDs	43	207.13	366.67	670.01	−2.105	0.035
The multiple CHDs	48	187.82	394.36	546.85	−2.006	0.045
The syndrome CHDs	6	115.05	252.31	670.01	−0.3300	0.742
PDA	70	174.64	378.04	653.72	−1.923	0.054
ASDs	48	201.79	400.54	521.04	−2.223	0.026
VSDs	7	242.17	480.73	670.01	−1.853	0.064
AVSDs	4	201.61	347.71	825.22	−0.608	0.543

### The concentration of titanium in the cord blood sample

The content of titanium in umbilical cord blood samples in the case group was higher than that in the control group, with statistical significance (*P* < 0.05), as showed in Table 3. The median titanium concentration in the cord blood was 320.07 μg/L, including the case group and the control group. Using the same method, we also found that there were statistically significant differences between some subtypes in the case group and the control group, such as The isolated CHDs, PDA, ASDs and VSDs (*P* < 0.05).

**Table 3:** Comparison of titanium content in umbilical cord blood between case group and control group.

**Umbilical cord serum titanium**						
**content [P**_**50**_ **(P**_**25**_**, P**_**75**_**)**, μ**g/L]**						
**Group**	**N**	**P_25_**	**P_50_**	**P_75_**	**Z**	***P*-value**
Control	194	246.37	316.84	393.65		
Case	97	264.86	370.43	459.76	−3.059	0.002
The isolated CHDs	43	276.72	457.33	570.41	−2.632	0.008
The multiple CHDs	48	261.39	347.47	460.50	−1.815	0.069
The syndrome CHDs	6	329.57	457.33	457.33	−1.512	0.131
PDA	70	259.82	366.46	458.86	−2.345	0.019
ASDs	48	261.94	357.56	481.24	−2.064	0.039
VSDs	7	381.27	457.33	618.32	−2.886	0.004
AVSDs	4	268.17	533.93	785.62	−1.032	0.302

### Relationship between serum titanium content in pregnant women and that in the umbilical cord

**Figure 2:**
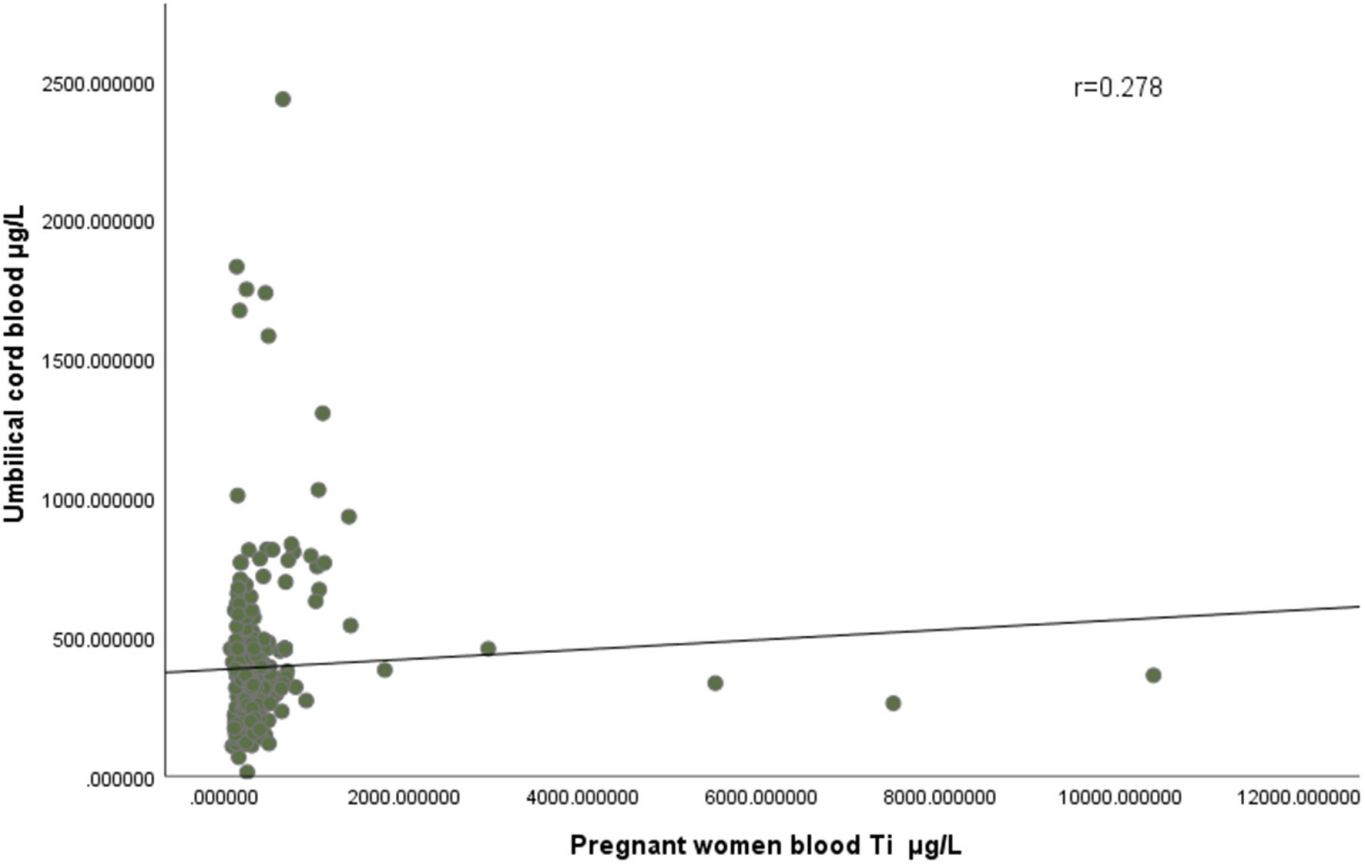
There was a positive correlation between the concentration of titanium in pregnant women's blood and that in umbilical cord blood (r = 0.278).

The maximum concentration of titanium in the blood of pregnant women in the case group and the control group was 10,366.45 μg/L and 1,384.84 μg/L, respectively, and the minimum concentration was 64.72 μg/L and 84.14 μg/L. The maximum concentration of titanium in the cord blood of the case group and the control group was 2,434.01 μg/L and 1,831.69 μg/L, respectively, and the minimum concentration was 116.93 μg/L and 13.90 μg/L, respectively. As showed in Figure 2, correlation analysis showed that there was a positive correlation between the concentration of titanium in pregnant women's blood and that in umbilical cord blood (r = 0.278).

### ROC curve analysis

ROC curve analysis showed that the Area Under Curve (AUC) area of maternal blood titanium level on the occurrence of CHDs was 0.589 (95% CI: 0.513–0.666, *P* = 0.013), and that of umbilical cord blood titanium level was 0.610 (95% CI: 0.539–0.681, *P* = 0.002). The cut-off values of titanium levels in maternal blood and umbilical cord blood were 404.22 μg/L and 453.58 μg/L, respectively.

### Association between serum titanium levels in pregnant women and CHDs in offspring

The association between different titanium levels and the risk of CHDs was further analyzed by dividing all maternal blood samples into low and high groups using cut-off values. As shown in Table 4, we found an increased risk of CHDs (aOR, 2.706, 95% CI, 1.547–4.734; *P* < 0.05), The multiple CHDs (aOR, 2.382, 95% CI, 1.219–4.655; *P* < 0.05), PDA (aOR, 2.412, 95% CI, 1.336–4.357; *P* < 0.05) and ASDs (aOR, 2.367, 95% CI, 1.215–4.609; *P* < 0.05) in the high concentrations of maternal blood titanium group in the model adjusted for all potential confounders. All results suggest that maternal blood titanium exposure may increase the risk of CHDs in offspring.

**Table 4:** Risks for fetal CHDs in different titanium in pregnant women blood concentrations.

**Group**	**Levels**	**Cases**	**Controls**	**cOR (95% CI)**	**aOR (95% CI)**
All CHDs	Low	51	151	Reference	Reference
	High	46	43	2.731 (1.589–4.694)	2.706 (1.547–4.734)
The isolated CHDs	Low	23	151	Reference	Reference
	High	20	43	1.820 (0.915–3.619)	1.976 (0.975–4.006)
The multiple CHDs	Low	24	151	Reference	Reference
	High	24	43	2.640 (1.384–5.035)	2.382 (1.219–4.655)
The Syndrome CHDs	Low	4	151	Reference	Reference
	High	2	43	0.777 (0.133–4.529)	0.829 (0.125–5.486)
PDA	Low	36	151	Reference	Reference
	High	34	43	2.512 (1.416–4.455)	2.412 (1.336–4.357)
ASDs	Low	24	151	Reference	Reference
	High	24	43	2.477 (1.297–4.730)	2.367 (1.215–4.609)
VSDs	Low	3	151	Reference	Reference
	High	4	43	2.165 (0.454–10.334)	3.430 (0.578–20.354)
AVSDs	Low	2	151	Reference	Reference
	High	2	43	1.908 (0.251–14.497)	1.462 (0.188–11.372)

aOR, adjusted odds ratio; cOR, crude odds ratio. Logistic regression was used to calculate odds ratios and 95% CIs; The optimal cut-off value of ROC curve of serum titanium concentration in pregnant women was divided into high and low concentrations; all models were adjusted for maternal pre-pregnancy BMI, maternal age, education, monthly household income, active/passive smoking during pregnancy (yes, no), alcohol consumption before pregnancy, take vitamins or minerals.

### Association between umbilical cord serum titanium content and CHDs in offspring

The association between different titanium levels and the risk of CHDs was further analyzed by dividing all umbilical cord blood samples into low and high groups using cut-off values. As shown in Table 5, we found an increased risk of CHDs (aOR, 3.129, 95% CI, 1.734–5.646; *P* < 0.05), The isolated CHDs (aOR, 2.922, 95% CI, 1.446–5.905; *P* < 0.05), The Syndrome CHDs (aOR, 11.834, 95% CI, 1.230–113.824; *P* < 0.05) and PDA (aOR, 2.207, 95% CI, 1.183–4.118; *P* < 0.05) in the high concentrations of umbilical cord blood group in the model adjusted for all potential confounders. All the results also indicated that the umbilical cord blood titanium exposure may increase the CHDs risk in offspring.

**Table 5:** Risks for fetal CHDs in different titanium in umbilical cord blood concentrations.

**Group**	**Levels**	**Cases**	**Controls**	**cOR (95% CI)**	**aOR (95% CI)**
All CHDs	Low	55	159	Reference	Reference
	High	42	35	2.996 (1.710–5.249)	3.129 (1.734–5.646)
The isolated CHDs	Low	21	159	Reference	Reference
	High	23	35	3.289 (1.661–6.511)	2.922 (1.446–5.905)
The multiple CHDs	Low	32	159	Reference	Reference
	High	16	35	1.222 (0.612–2.439)	1.362 (0.656–2.827)
The syndrome CHDs	Low	2	159	Reference	Reference
	High	4	35	6.105 (1.060–35.163)	11.834 (1.230–113.824)
PDA	Low	41	159	Reference	Reference
	High	29	35	2.168 (1.200–3.919)	2.207 (1.183–4.118)
ASDs	Low	29	159	Reference	Reference
	High	19	35	1.753 (0.897–3.428)	1.795 (0.894–3.606)
VSDs	Low	2	159	Reference	Reference
	High	5	35	6.277 (1.153–34.162)	4.781 (0.798–28.661)
AVSDs	Low	2	159	Reference	Reference
	High	2	35	2.463 (0.324–18.718)	2.916 (0.366–23.222)

aOR, adjusted odds ratio; cOR, crude odds ratio. Logistic regression was used to calculate odds ratios and 95% CIs; The optimal cut-off value of ROC curve of serum titanium concentration in pregnant women was divided into high and low concentrations; all models were adjusted for maternal pre-pregnancy BMI, maternal age, education, monthly household income, active/passive smoking during pregnancy (yes, no), alcohol consumption before pregnancy, take vitamins or minerals.

## Discussion

The findings of this study showed that the fetal cord blood titanium concentration in the CHDs group was higher than that in the control group. Our results indicate that increased umbilical cord and maternal blood titanium concentrations are significantly associated with CHDs risk. This is in line with epidemiological data, which suggests that greater levels of titanium in the umbilical cord and maternal blood may be linked to a higher risk of CHDs in offspring, including some major subtypes.

In our study, the median titanium concentration in pregnant women and the cord blood were 304.65 and 320.07 μg/L, respectively, including the case group and the control group. Compared with previous human studies, the value of titanium in our study is higher (26, 27), which probably due to the high titanium exposure of mining around Lanzhou. Widespread variations in blood titanium concentrations may result from changes in living conditions, dietary preferences, exposure to airborne particulate matter, and metal analysis techniques (28).

The placenta is an important organ responsible for the exchange of substances between the fetus and the mother, and is related to the health of the mother and the fetus. Compared to heavy metal exposure through fetal placental tissue (5), We used umbilical cord blood to analyze titanium exposure to give a more intuitive picture of the actual fetal titanium exposure level. A recent study confirmed the transmission of titanium across the placental barrier to the fetus during pregnancy by detecting titanium levels in meconium samples (29), this study also demonstrated that titanium can cross the placental barrier by detecting titanium levels in umbilical cord blood, and our results showed that fetal exposure to titanium increases with maternal exposure. In addition, animal experiments demonstrated that gestation exposure to titanium not only significantly impaired the growth and development of the placenta in mice, but also increased the total peripheral resistance of the placenta and impaired umbilical cord blood reactivity (30, 31). The mechanism of titanium induced placental dysfunction may be related to the reduction of formation of the placental vascular system (32), induction of autophagy (33) and endocrine disorders (12).

When titanium can cross the placental barrier and enter the fetus, it may affect the developing heart. It has been suggested that the RA signal caused by TiO_2_ NPs may affect the developing heart of the fetus (12). According to studies, prenatal exposure to TiO_2_ NPs impaired mitochondrial metabolism by reducing the electronic transmission chain (ETC) in the progeny (34). The decreased mitochondrial ETC can also limit the contractility of myocardial cells and harm the progeny's heart function and bioenergetics (35, 36). Pan et al. use of quantitative proteomics approach applying iTRAQ-based mass spectrometry (MS) analysis found that TiO_2_ antiparticle may cause human embryonic stem cells (hESCs) to lose their pluripotency and disrupt the differentiation of hESCs mesoderm into cardiomyocytes, thus affecting the normal development of cardiomyocytes, and it was also found that this may be due to DNA damage and oxidative stress in hESCs after TiO2 exposure (37). Therefore, minimizing titanium exposure during pregnancy is essential.

Our study has a few limitations. First of all, the questionnaires and samples in this study were collected 10 years ago, representing the exposure level of titanium in the environment at that time, which may have some deviation from the current exposure situation. Second, we only measured titanium concentrations at one point in the third trimester of pregnancy and could not assess changes in titanium levels during pregnancy. Third, the interaction between titanium and other heavy metal was not considered in this study. Further studies are necessary to confirm these findings.

## Conclusion

Overall, titanium could cross the placental barrier, and fetal titanium exposure was significantly higher in the CHDs group than in the control group. Titanium during pregnancy was associated with CHDs risk at a high level of exposure. Further research is needed to investigate the underlying mechanism of congenital heart disease caused by titanium exposure during pregnancy.

## Data availability statement

The raw data supporting the conclusions of this article will be made available by the authors, without undue reservation. Requests to access the data sets should be directed to 1038817191@qq.com.

## Ethics statement

This study was approved by the Institutional Ethics Committee of the Gansu Provincial Maternity and Child-care Hospital, China (2012-5). Written informed consent was obtained from all participants for their participation in this study.

## Author contributions

JS, BM, and ZW: conceptualization, formal analysis, software, and writing—original draft. XJ: investigation and formal analysis. YW: writing—review and editing. YL and XM: data curation and validation. XLiu and XLin: methodology. XX: investigation. HC: resources. JQ: project administration. BY: funding acquisition. QL: conceptualization and supervision. All authors contributed to the article and approved the submitted version.

## Funding

This work was supported in part by the Gansu Provincial Science and Technology Department Grant (No. 21JR1RA043), the Key Research and Development Program of Gansu Province (No. 20YF8WA095), and the Coronavirus disease prevention and control research program of Lanzhou city (2020-XG-12).

## Conflict of interest

The authors declare that the research was conducted in the absence of any commercial or financial relationships that could be construed as a potential conflict of interest.

## Publisher's note

All claims expressed in this article are solely those of the authors and do not necessarily represent those of their affiliated organizations, or those of the publisher, the editors and the reviewers. Any product that may be evaluated in this article, or claim that may be made by its manufacturer, is not guaranteed or endorsed by the publisher.
